# Optimization of PID control parameters for marine dual-fuel engine using improved particle swarm algorithm

**DOI:** 10.1038/s41598-024-63253-y

**Published:** 2024-06-03

**Authors:** Zhuo Hu, Weihao Guo, Kege Zhou, Lei Wang, Fu Wang, Jinliang Yuan

**Affiliations:** 1 Faculty of Maritime and Transportation, Ningbo University, Ningbo, 315211 People’s Republic of China; 2 China Coal Society, Beijing, 100013 People’s Republic of China

**Keywords:** Dual-fuel engine, Particle swarm algorithm, PID control, Air–fuel ratio, Fuel replacement ratio, Marine biology, Natural gas

## Abstract

This study presents a comprehensive investigation into the optimization of PID control parameters for marine dual-fuel engines using an improved particle swarm algorithm. Through the development of a Matlab/Simulink simulation model, the thermodynamic behavior of the engine and the functionality of its control system are analyzed. The PID control parameters for air–fuel ratio control and mode switching control systems are fine-tuned utilizing the improved particle swarm algorithm (PSO). Simulation results demonstrate that the proposed improved PID-PSO approach outperforms traditional PID and traditional PSO-PID control methods in terms of reduced overshoot, minimized steady-state error, faster response times, and improved stability across various operating conditions and response modes. In comparison to traditional PID and PSO-PID controllers, the improved PSO-PID controller reduces the response time by 0.47 s and 0.21 s, the maximum overshoot by 98.43% and 96.05%, and decreases the absolute errors by 87.42% and 90.55%, respectively, in air–fuel ratio control using the step response method. The study's findings offer valuable insights into enhancing the performance and efficiency of marine dual-fuel engines through advanced control strategies.

## Introduction

With the increasing global concern for environmental protection and energy sustainability, the maritime industry is actively seeking more environmentally friendly and economical power solutions^1^, using clean energy to achieve decarbonization is also the theme of the times^2–4^ In this context, dual-fuel marine engines have attracted considerable attention due to their advantages in reducing emissions and improving fuel efficiency^5^. Dual-fuel engines can simultaneously utilize traditional liquid fuels and clean alternative fuels such as liquefied natural gas (LNG) to achieve more environmentally friendly and economical vessel operations^6^.

However, the control of marine dual-fuel engines faces a series of challenges, notably in air–fuel ratio control and mode switching^7^. Precise control of air–fuel ratio control is crucial to ensure stable combustion efficiency and emission performance across varying operating conditions^8^. Mode switching control involves seamless transitioning between liquid and gas fuels to meet diverse operating demands^9^. Although dual-fuel engines use both liquid fuel and clean energy sources like LNG, the significant differences in their characteristics, including ignition properties, energy density, and supply system requirements, necessitate intricate adjustment in the air–fuel ratio to ensure optimal combustion efficiency and performance stability across different fuel modes^10^. Furthermore, the dynamic nature of sea conditions and load variations during navigation introduces dynamic fluctuations in engine operating conditions, posing challenges in maintaining air–fuel ratio stability. Addressing these challenges requires real-time adjustments in fuel supply and ignition control to accommodate varying conditions effectively^11^. Transitioning between liquid fuel and LNG modes may occur complications such as unstable fuel supply or ignition delays, culminating in performance degradation and heightened emissions^12^.

Air–fuel ratio control and mode switching of dual-fuel engines involve multiple subsystems, including fuel supply systems, ignition systems, and control logic, necessitating sophisticated control algorithms and reliable hardware to ensure the coordinated operation and reliability of these subsystems^13^. To enhance engine performance, numerous studies have delved into engine control methodologies. PID controller is widely used because of its simple structure and good robustness^14^. Toriki et al.^15^ proposed an air–fuel ratio control system for dual-fuel engines employing PID control. Given that the parameters of the traditional PID controller are fixed, it is unable to make real-time adjustments for nonlinear factors, making it difficult to further improve the control performance. Nonlinear modification of control parameters in traditional PID controllers can reduce or even overcome the influence of these nonlinear factors^16^. Nonetheless, nonlinear PID controllers require tuning of more parameters, complicating the process. Furthermore, these methods are not globally convergent, making it difficult to ensure optimal control effects.

On this basis, more and more research has begun to turn towards the development of advanced control methods. These advanced methods aim to address the limitations of traditional PID controllers by enhancing their ability to handle nonlinear factors and improve overall control performance. Techniques such as adaptive control, fuzzy logic control, and machine learning-based optimization are being explored to provide more dynamic and precise adjustments in real-time. For instance, Najm et al.^17^ utilized a genetic algorithm (GA) to tune the parameters of a nonlinear PID controller in unmanned aerial vehicle systems. This approach leverages the GA’s ability to efficiently search for optimal solutions across complex, multi-dimensional spaces, thereby enhancing the performance of the PID controller in handling nonlinearities and dynamic changes in the system. However, the genetic algorithm requires a large number of samples for solution, incurs a high time cost, and may fall into local optimal solutions. Pathak et al.^18,19^ used an artificial gorilla force optimizer (GTO) to obtain controller parameters. The results show that the method has the advantages of small frequency deviation, short stability time and small integration error. Wang et al.^20^ explored the optimization of air–fuel ratio control in fuel engines through adaptive fuzzy PID. Despite these efforts, fuzzy PID control exhibits drawbacks such as low precision, diminished stability, susceptibility to disturbances, and protracted adjustment times. Shu et al.^21^ integrated PID control with the cuckoo search algorithm to manage mode switching in dual-fuel engines. Although the cuckoo search algorithm offers simplicity and ease of implementation with fewer parameters, it suffers from slow search speeds and relatively low precision. Particle swarm optimization (PSO) methods have emerged as a solution to address challenges associated with expert knowledge and control evaluation in describing PID controllers^22^. Researchers have applied PSO mathematical theory to optimize PID control parameters^23^, leading to stable control of engine air–fuel ratio and speed in simulation studies. However, traditional PSO algorithms are plagued by issues such as premature convergence and slow convergence speeds, hindering the achievement of high-precision control effects^24^. In addition, most existing improvements primarily focus on overshoot and response speed. However, dual-fuel engine systems demand high control accuracy and faster real-time control speeds. Therefore, a more efficient control strategy for controller optimization is essential to accelerate system response speed, suppress overshoot, and achieve high steady-state accuracy.

In addressing the nonlinearity and time-varying nature of large-scale marine dual-fuel engine systems with complex models, as well as the limitations of traditional PSO algorithms, cuckoo algorithm and GTO in tuning PID parameters, this paper proposes a PID controller optimization based on improved particle swarm optimization algorithm to optimize the control of air–fuel ratio and mode switching. The proposed method not only addresses the issues of premature convergence and local optimal search commonly encountered in traditional PSO algorithms but also mitigates the challenges of slow search speed and low accuracy associated with methods like the cuckoo algorithm and fuzzy algorithm. By dynamically adjusting the inertia weight based on similarity, the improved PSO algorithm can achieve a better balance between exploration and exploitation. To evaluate its effectiveness, the method is applied to a simulation of a dual-fuel engine model developed in the Matlab/Simulink environment. Comparative analysis is conducted with traditional PID control and standard PSO-PID control to assess the performance of the controllers in air–fuel ratio control and mode switching scenarios.

The main contributions of this paper is presented briefly as:

1. Proposal and modeling of a sophisticated large-scale dual-fuel engine model based on the Matlab/Simulink environment.
2. Introduction of an improved PSO algorithm to optimize the parameters of the traditional PID controller.
3. Designing an effective similarity inertia weight function to ensure fast system response speed, low overshoot, and small steady-state errors.
4. Air–fuel ratio and mode switching of the dual-fuel engine, solving issues of low optimization control precision, slow speed, and local optimal solutions.
5. Insights for controling the dual-fuel engine for actual response modes in marine application.

## Model of dual-fuel engine

**Figure 1:**
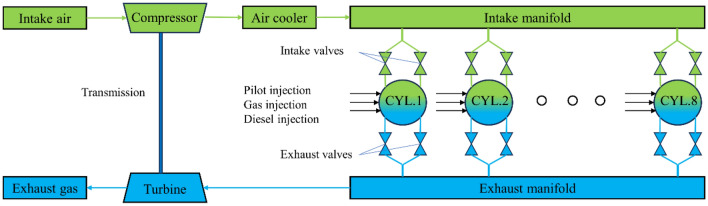
Structure and working principle of marine dual-fuel engine.

Natural gas engines can be classified into two basic types based on the combustion method: single-fuel engines using spark plug ignition and dual-fuel engines that primarily use natural gas as the main fuel with high-reactivity fuels such as diesel as pilot fuel. The inclusion of pilot fuel in the latter addresses the challenge of ignition difficulty, especially under high fuel replacement rates, ensuring smoother and more reliable engine operation. In dual-fuel engines for vehicles, two main natural gas injection technologies are employed: intake manifold pre-mixed injection and in-cylinder direct injection. There are three popular types of dual-fuel engines for marine applications: two-stroke high-pressure injection, two-stroke low-pressure injection, and four-stroke intake manifold injection^25^. Figure 1 illustrates the main structure of the marine dual fuel engine. The model comprises key components such as the intake and exhaust modules, fuel supply module, cylinder module, supercharger module, and air cooler module for dual-fuel engines. Table 1 presents the main technical specifications of the MAN 8L51/60DF dual-fuel engine^26^.

**Table 1:** Main technical parameters of marine dual-fuel engine.

Parameter	Unit	Value
Number of cylinders	–	8
Diameter	mm	510
Stroke	mm	600
Compression ratio	–	13.3
Power rating	kW	8000
Rated speed	r/min	514
Fuel consumption	g/kWh	189.1
Effective pressure	bar	19.1
Firing order	–	1–4–7–6–8–5–2–3

### Basic equation for thermodynamics

The operation of dual fuel engine involves a series of complex chemical combustion, physical transmission, gas/liquid flow and heat transfer processes, which can be simplified by the following equations.

Energy conservation equation:

1 $$dU = dW + \sum d Q_{i} + \sum\limits_{j} {h_{j} } \cdot dm_{j}$$

where *h*_*j*_⋅*dm*_*j*_ is the energy entering or exiting the system, kJ; *Q*_*i*_ is the heat exchanged through the system, kJ; *W* is the work exerted on the piston by the expansion of the gas, kJ; *h*_*j*_ is the enthalpy, kJ/kg; *U* is the internal energy of the system, kJ.

Conservation of mass equation:

2 $$\frac{dm}{{d\varphi }} = \frac{{dm_{s} }}{d\varphi } + \frac{{dm_{e} }}{d\varphi } + \frac{{dm_{B} }}{d\varphi }$$

where *m*_*e*_ is the outflow cylinder mass, kg; *m*_*B*_ is the instantaneous fuel mass in the cylinder, kg; *m*_*s*_ is the inflow cylinder mass, kg.

Ideal gas equation of state:

3 $$pV = mRT$$

where* p* is the pressure, MPa; *V* is the volume,* T* is the temperature, K;* R* is the gas constant, kJ/(kg^.^K).

### Cylinder mathematical model

The cylinder module stands out as the most intricate and pivotal component within the entire engine modeling framework. The modeling of cylinder module comprises three aspects: the cylinder working volume, combustion, and cylinder perimeter wall heat transfer. This module plays a crucial role in capturing and simulating the complex dynamics occurring within the engine cylinder^27^. The cylinder working volume and volume change rate can be presented as follows:

4 $$Vs = \frac{{\pi D^{2} }}{4}\left\{ {\frac{S}{\varepsilon - 1} + \frac{S}{2}\left[ {\left( {1 + \frac{1}{\lambda }} \right) - \cos \left( {\frac{\pi }{180}\varphi } \right) - \frac{1}{\lambda }\sqrt {1 - \lambda^{2} \sin^{2} \left( {\frac{\pi }{180}\varphi } \right)} } \right]} \right\}$$

5 $$\frac{dVs}{{d\varphi }} = \frac{{\pi^{2} D^{2} S}}{8 \times 180}\left[ {\sin \left( {\frac{\pi }{180}\varphi } \right) + \frac{\lambda }{2} \cdot \frac{{\sin \left( {\frac{\pi }{180} \cdot 2\varphi } \right)}}{{\sqrt {1 - \lambda^{2} \sin^{2} \left( {\frac{\pi }{180}\varphi } \right)} }}} \right]$$

where *V*_*s*_ is cylinder working volume, L; *λ* is connecting rod to crank ratio; *φ* is crankshaft angle; *S* is fuel injection penetration; *D* is the diameter of the jet hole; *ε* is compression ratio.

In the context of non-predictive combustion models, the combustion rate is solely influenced by the crankshaft rotation angle, irrespective of whether the cylinder's operating conditions meet the combustion demand. Given the substantial impact of air–fuel ratio and fuel substitution rate on the combustion rate, coupled with the direct influence of injection parameter settings on the combustion process within the cylinder, the MAN 8L51/60DF dual-fuel engine employed in this study integrates the predictive combustion model DI-Pluse from Matlab/Simulink. This choice facilitates an exploration of the effects of these variables on power output, fuel consumption, and exhaust emissions in a dual-fuel engine. Distinct thermodynamic state parameters, compositions, and concentrations characterize the discrete thermodynamic partitions, and their independence from one another is a notable feature. The primary unburned zone, or first partition, comprises gases remaining in the cylinder after closure. The second and third partitions consist of the injected unburned zone-composed of inhaled fuel and gases-and the injected burned zone, representing combustion products. The mathematical model used to simulate this process is as follows.

Divided length:

6 $$L_{b} = 0.7D\left( {1 + 0.4\frac{r}{D}} \right)\left( {\frac{{\rho_{g} }}{{\rho_{l} v_{i}^{2} }}} \right)^{0.05} \left( \frac{L}{D} \right)^{0.13} \left( {\frac{{\rho_{l} }}{{\rho_{g} }}} \right)^{0.5}$$

where* L* is the length of the jet hole; *v*_*i*_ is the jet velocity;* ρ*_*l*_ is the density of the injected fuel; and *ρ*_*g*_ is the density of the surrounding gas.

Angle of the spray cone:

7 $$\theta = 83.5\left( \frac{L}{D} \right)^{ - 0.22} \left( {\frac{D}{{D_{0} }}} \right)^{0.15} \left( {\frac{{\rho_{g} }}{{\rho_{1} }}} \right)^{0.26}$$

Fuel injection penetration:

8 $$S = 0.39\left( {\frac{2\Delta p}{{\rho_{l} }}} \right)^{0.5} t,\quad 0 < t < t_{b}$$

9 $$S = 2.95\left( {\frac{2\Delta p}{{\rho_{l} }}} \right)^{0.25} (Dt)^{0.5} ,\quad t > t_{b}$$

10 $$t_{b} = 28.65\frac{{\rho_{1} D}}{{\left( {\rho_{g} \Delta p} \right)^{0.5} }}$$

where* t*_*b*_ is the oil beam splitting time; $\Delta$*p* is the jet pressure difference; *D*_*0*_ is initial diameter of spray hole.

Fire delay duration:

11 $$I = \int {\frac{1}{\tau }} dt$$

12 $$\tau = ap^{b} \varphi^{c} \exp (d/Z)$$

where *a* is the ignition delay factor; *b* is the pressure delay exponent; *c* is the empirical constant; *d* is the delayed activation temperature; *Z* is the ignition delay period; *φ* is the equivalence ratio.

Rates of combustion and volumetric absorption can be defined as:

13 $$\frac{{dm_{e} }}{dt} = \sum\limits_{j} {\left( {\rho_{f,j} ,\rho_{a} } \right)^{1/2} } 2\pi R_{j} \left( {x_{j + 1} - x_{j} } \right)C_{x} \left| {v_{j} - v_{a,j} } \right|$$

14 $$\frac{{dm_{B} }}{dt} = C_{1} \frac{{m_{c} }}{{\tau_{c} }}$$

15 $$\tau_{c} = \frac{{l_{T} }}{{S_{L} }}$$

where *x*_*j*_ is the coordinate of the* j*th cell along the spray axis; *R*_*j*_ is the radius of the* j*th cell; *v*_*j*_ is the velocity of the local jet; *v*_*a, j*_ is the velocity of the air; *C*_*x*_ is the empirical constant; *S*_*L*_ is the laminar flame velocity.

Diffusion combustion models for computing volumetric absorption and burning rates:

16 $$\frac{{dm_{e} }}{dt} = C_{e} \frac{{m_{e} }}{{\tau_{e} }}$$

17 $$\tau_{e} = \left( {\frac{{l_{I} }}{\varepsilon }} \right)^{1/3}$$

18 $$l_{I} = C_{\mu }^{3/4} \frac{{k^{3/2} }}{\varepsilon }$$

19 $$\frac{{dm_{B} }}{dt} = \frac{{m_{e} }}{{\tau_{c} }}$$

where *l*_*I*_ is the integrated length scale, *C*_*u*_ is 0.09, and *C*_*e*_ is the pre-combustion coefficient before the ignition point of each partition, *k* is insulation index.

Heat exchange occurs throughout the entire operation of the engine, such as between the external environment and the cylinder body, as well as between the cylinder body and the cylinder mixture. Heat losses are calculated through a heat transfer model, which transforms the complex heat transfer processes into a heat transfer coefficient model. The Woschni, Eichelberg, Annand, and Sitkei formulas can all be used to calculate the instantaneous heat transfer coefficient. In this paper, the Woschni heat transfer model is employed to calculate the heat transfer coefficient. This model is widely used in the field of internal combustion engine simulation modeling due to its simplicity and accuracy. The calculation formula is as follows^28^:

20 $$\alpha_{g} = 820 \cdot D^{ - 0.2} \cdot P_{z}^{0.8} \cdot T_{z}^{ - 0.53} \left[ {c_{1} \cdot c_{m} + c_{2} \cdot \frac{{V_{s} T_{1} }}{{P_{1} V_{1} }}\left( {P_{z} - P_{o} } \right)} \right]^{0.8}$$

where* P*_*z*_ is the instantaneous cylinder pressure, MPa; *T*_*z*_ is the instantaneous cylinder temperature, K; *P*_*1*_, *T*_*1*_, *V*_*1*_ is the pressure, temperature, and volume at the starting point of compression; *c*_*m*_ is the average piston velocity, m/s; *c*_*1*_ is the gas velocity coefficient; *c*_*2*_ is combustion chamber shape coefficient.

### Intake and exhaust mathematical model

Due to the influence of exhaust gas pressure fluctuations on the efficiency of the turbocharger and the residual gas quantity in the cylinder, and the direct impact of intake manifold pressure fluctuations on the intake volume within the cylinder, it is necessary to perform detailed calculations of the gas flow in the dual fuel engine’s intake and exhaust pipes.

In the simulation and modeling calculations, it is common to simplify the three-dimensional non-isotropic flow simulation of the dual fuel engine's intake and exhaust processes into an unsteady one-dimensional flow. During the engine's operation, the relevant parameters of the intake and exhaust systems are constantly changing. The pipe diameter is much smaller than the pipe length, resulting in radial flow dominating the entire flow process. Therefore, the intake and exhaust processes are treated as one-dimensional, and the simulation calculation method adopts the one-dimensional finite volume method.

Firstly, it is necessary to establish the differential equations relating the state parameters such as velocity, temperature, and pressure of the pipe segment to the corresponding crankshaft rotation angle and axial direction. The solution of the continuity equation, energy equation, and momentum equation are crucial for the mathematical model of the intake and exhaust systems.

The continuous equation is presented as:

21 $$\frac{dm}{{dt}} = \sum {m_{in} } = \sum \rho Au$$

where *m*_*in*_ is the inlet mass flow rate, kg/s; *m* is the mass of the work fluid in the boundary, kg; *ρ* is the density of the fluid in the pipe, kg/m^3^. *A* is the corresponding cross-sectional area of the pipe, m^2^; *u* is the velocity of the control body boundary, m/s.

The energy equation is expressed:

22 $$\frac{d(me)}{{dt}} = p\frac{dV}{{dt}} + \sum {\left( {m_{in} \cdot H} \right)} - hA_{s} \left( {T_{{\text{fluid }}} - T_{{\text{wall }}} } \right)$$

where *A*_*s*_ is the heat transfer surface area of the inner wall of the tube, m^2^; *p* is the pressure, MPa; *H* is the total enthalpy corresponding to the fluid, J/kg; *h* is the heat transfer coefficient, W/(m^2.^K); *T*_*fluid*_ is the fluid temperature, K; *T*_*wall*_ is the wall temperature of the tube wall, K.

### Turbocharger mathematical model

In a waste gas turbocharged engine, the waste gas turbine converts the energy from the engine's exhaust gases into mechanical work through a turbine. This mechanical work is then transferred to the compressor, which compresses the air to increase the volume of air entering the engine and raise the intake pressure. Throughout this process, the turbine and compressor must achieve power balance, speed balance, and flow balance^29^.

The power balance is expressed as:

23 $$W_{K} = W_{T} \cdot \eta_{TKm}$$

where *W*_*K*_ is the work consumed by the compressor, kW; *W*_*T*_ is the turbine work, kW; and *η*_*TKm*_ is the supercharger efficiency.

The rotational speeds of the compressor, turbine and supercharger are the same:

24 $$n_{K} = n_{T} = n_{TK}$$

where *n*_*K*_ is the speed of the compressor; *n*_*T*_ is the speed of the turbine; and *n*_*TK*_ the shaft speed of the supercharger.

The exhaust gas flow rate is the sum of the air and fuel flow rate:

25 $$\dot{m}_{T} = \dot{m}_{K} + \dot{m}_{f}$$

where $\dot{m}_{T}$ is the exhaust gas flow rate; $\dot{m}_{K}$ is the air flow rate; and $\dot{m}_{f}$ is the fuel mass flow rate.

The exhaust gas turbine plays a crucial role in powering the supercharger, and its model should encompass key features such as air flow, boost ratio, speed, and efficiency. The supercharger model, in turn, is characterized by two input parameters: air flow rate and supercharger speed, while the output parameters include temperature (*T*_*o*_) and outlet pressure (*P*_*o*_).

The pressurization ratio and the turbine's rotor speed can be calculated using the principles of thermodynamics and Newton's laws. Specific flow rate and efficiency data for the compressor can be acquired by referring to relevant tables. Subsequently, the compressor's efficiency can be determined using the one-dimensional isentropic adiabatic flow theory. Finally, the outlet temperature (*T*_*o*_) and outlet pressure (*P*_*o*_) can be calculated using the one-dimensional isentropic adiabatic compression process, with the arithmetic formula provided as follows.

26 $$P_{o} = P_{i} \pi_{c}$$

27 $$T_{o} = T_{e} \left\{ {1 + \frac{1}{{\eta_{c} }}\left[ {\pi_{c} \left( {\frac{k - 1}{k}} \right) - 1} \right]} \right\}$$

where *P*_*i*_ is the atmospheric pressure, Pa; *T*_*e*_ is the environmental temperature, K.

The pressurization ratio and isentropic efficiency of the compressor are contingent upon both the air flow rate and the rotational speed of the compressor. These functional dependencies can be expressed through a mathematical relationship:

28 $$\pi_{c} = f\left( {n_{c} ,q_{c} } \right)$$

29 $$\eta_{c} = f\left( {n_{c} ,q_{c} } \right)$$

The above functional relationships are typically determined based on characteristic curves of a specific model of a turbocharged gas engine. Due to the absence of characteristic curves for the turbocharger, a modeling approach is adopted using the characteristic curves of a diesel engine. Specifically, the pressurization ratio and efficiency of the turbocharger are formulated as a one-dimensional table that varies with the load of the dual-fuel engine, represented using the one-dimensional lookup table module in Simulink. The modeling process of the turbocharger involves the utilization of additional structural or performance parameters, with specific numerical values as detailed in Table 2.

**Table 2:** Parameters related to superchargers.

Parameter name	Unit	Parameter value
Pressurizer inlet pressure	MPa	0.103
Pressurizer inlet temperature	K	303
Gas insulation index	–	1.4
Gas constant	–	287

### Intercooler model

The intercooler serves to cool the fresh air entering the cylinder, thereby improving the turbocharging efficiency of the engine. The input parameters for the intercooler model include the air temperature, air pressure, and air mass flow rate at the outlet of the turbocharger. As air flows through the intercooler, there is a pressure loss and temperature decrease. By introducing the intercooler effectiveness coefficient (i.e., the temperature at the outlet of the intercooler), the outlet temperature of the intercooler is calculated using a simplified method. By introducing the intercooler effectiveness coefficient (*β*) and combining it with experimental data, the outlet temperature of the intercooler is calculated using a simplified method, with *β* set to 0.88, resulting in the intercooler outlet temperature.

30 $$T_{j} = T_{o} (1 - \beta ) + \beta T_{w}$$

where *T*_*w*_ is cooling water inlet temperature, K; *β* is cooling coefficient is generally taken as 0.7–0.9.

Intercooler pressure loss *P*_*s*_ is defined as:

31 $$P_{s} = P_{s0} \left( {\frac{{q_{m} }}{{q_{m0} }}} \right)$$

where *q*_*m*_ is actual flow rate of the intercooler, kg/s; *P*_*s0*_ is pressure loss of the intercooler at design operating conditions, Pa; *q*_*m0*_ is intercooler design flow rate, kg/s.

Then the air pressure at the outlet is:

32 $$P_{r} = P_{o} - P_{s}$$

The values of the intercooler parameters are shown in Table 3.

**Table 3:** Values of intercooler related parameters.

Parameter	Unit	Value
Intercooler cooling efficiency	–	0.88
Cooling water inlet temperature	K	295
Intercooler design pressure loss	Pa	300

### Cylinder intake model

The cylinder charging coefficient is a parameter that quantifies the amount of fresh air remaining in the cylinder at the end of the intake stroke relative to the volume of the intake manifold. Research results indicate that there is a functional relationship between the cylinder charging coefficient and the engine speed. Therefore, this article uses the least squares method to fit the engine speed and boost coefficient data to obtain the boost coefficient curve:

33 $$\eta_{v} = a_{0} + a_{1} \times n + a_{2} \times n^{2}$$

where *a*_*0*_, *a*_*1*_, *a*_*2*_ are the fitted constants.

Since the model established is an average model for studying air–fuel ratio control methods, it overlooks the residual exhaust gases in the cylinder after each engine cycle. According to the ideal gas equation, the intake air flow can be calculated as:

34 $$q_{m1} = \frac{{\eta_{v} nVsP_{r} }}{{120RT_{j} }}$$

### Fuel sub-model

For the MAN 8L51/60DF LNG/diesel dual-fuel engine, a fuel sub-model is established based on the propulsion characteristics. Under propulsion characteristics, the relationship between power and speed is given by:

35 $$P = F \cdot n^{3}$$

where *P* is engine power, kW; *F* is scale factor.

The corresponding values of propulsion characteristic speed and load are shown in Table 4.

**Table 4:** Characteristic speed and load corresponding values during propulsion.

n (r/min)	303	388	447	514
Load (%)	25	50	75	100

To accurately establish the fuel supply sub-model for marine liquefied natural gas/diesel dual-fuel engines, it is necessary to explore the fuel injection quantity under propulsion characteristics^30^. In dual-fuel engines, both diesel and natural gas are introduced into the engine cylinder for combustion. The calculation necessitates the incorporation of the substitution ratio, which denotes the reduction in the amount of diesel consumed by the engine in dual-fuel operating mode compared to the consumption in pure diesel operating mode^31^. This ratio is expressed as a percentage of the diesel consumption in pure diesel operating mode^32^.

36 $$T_{m} = \frac{{B - B_{o} }}{B} \times 100\%$$

where *T*_*m*_ is diesel replacement rate, %; *B* is diesel consumption in diesel-only mode, kg/h; *B*_*o*_ is diesel consumption in dual-fuel mode, kg/h.

The natural gas entering the cylinder precisely substitutes the reduced diesel fuel in the combustion process, and the substitution rate equals the doping rate. The calculation of the substitution rate is as follows^33^. Under specific operating conditions and at a specific substitution rate, the cylinder injection volume per cycle and the natural gas injection volume are determined based on theoretical considerations.

37 $$\left\{ {\begin{array}{*{20}l} {H_{ul} \cdot B_{l} = \left( {B - B_{o} } \right) \cdot H_{uo} } \hfill \\ {\frac{{B - B_{o} }}{B} = T_{m} } \hfill \\ \end{array} } \right.$$

where *B*_*l*_ is natural gas consumption in dual fuel mode, g/cycle; *H*_*ul*_ is low calorific value of natural gas, MJ/kg; *H*_*uo*_ is low calorific value of diesel, MJ/kg.

Based on the propulsion characteristics, the circulating injection volume and natural gas injection volume are calculated under various working conditions and different fuel substitution rates, following the aforementioned calculation process. Some of the calculation results are presented in Table 5.

**Table 5:** Partial cyclic injection volume and natural gas injection volume.

Load (%)	25	25	40	40	50	50	75
Replacement rate (%)	10	30	30	50	50	70	30
Fuel injection (g/cycle)	20.792	16.172	21.619	15.442	17.181	10.309	30.537
Gas injection (g/cycle)	1.901	5.743	7.684	12.807	14.261	19.965	10.872

### Emissions calculation

The calculation of NO_X_ and HC emissions is as follows:

38 $${\text{MNO}}_{{\text{x}}} = {\text{NO}}_{{\text{x}}} {\text{W}} \times \left[ {\begin{array}{*{20}l} {0.001586 \times (1 - Tm) + \left( {\frac{{{\text{CH}}_{4} }}{100} \times 0.001621 + \frac{{{\text{C}}_{3} {\text{H}}_{8} }}{100} \times 0.001603} \right.} \hfill \\ {\left. { + \frac{{{\text{C}}_{4} {\text{H}}_{10} }}{100} \times 0.001600} \right) \times Tm} \hfill \\ \end{array} } \right] \times \frac{{\dot{m}_{T} }}{1000}$$

39 $${\text{MHC}} = {\text{HCW}} \times \left[ {\begin{array}{*{20}l} {0.000479 \times (1 - Tm) + \left( {\frac{{{\text{CH}}_{4} }}{100} \times 0.000558 + \frac{{{\text{C}}_{3} {\text{H}}_{8} }}{100} \times 0.000512} \right.} \hfill \\ {\left. { + \frac{{{\text{C}}_{4} {\text{H}}_{10} }}{100} \times 0.000505} \right) \times Tm} \hfill \\ \end{array} } \right] \times \frac{{\dot{m}_{T} }}{1000}$$

where NO_X_W represents the wet volume concentration of nitrogen oxides in parts per million (ppm), HCW denotes the wet volume concentration of hydrocarbons in parts per million (ppm), MNO_X_ signifies the mass flow rate of nitrogen oxides in grams per kilowatt-hour (g/kWh), and MHC denotes the mass flow rate of hydrocarbons in grams per kilowatt-hour (g/kWh). The detailed calculation and derivation of these parameters can be found in literature^34^. Table 6 provides information on the main components of the fuel along with their respective proportions.

**Table 6:** Main composition of the fuel and their respective proportions.

Diesel		Natural gas	
C/%	86.74	CH_4_/%	92.682
H/%	12.95	C_2_H_2_/%	4.175
N/%	0.14	C_3_H_8_/%	0.184
O/%	0.04	C_4_H_10_/%	0.224
S/%	0.06	CO_2_/%	1.723

## PID control based on improved particle swarm algorithm

### PID controller principle

The PID controller, being a second-order linear controller, is influenced by its proportional, integral, and differential parameters, allowing for direct modification of its control impact by adjusting these parameters^35^. The most commonly used PID controllers operate based on the following principles^36^:

40 $$u(h) = K_{p} \left[ {e(h) + \frac{1}{{T_{i} }}\int_{0}^{h} e (h){\text{d}}h + \frac{{T_{d} {\text{d}}e(h)}}{{{\text{d}}h}}} \right]$$

Expressed by transfer function as:

41 $$G(s) = \frac{U(s)}{{E(s)}} = K_{p} + \frac{{K_{i} }}{S} + K_{d} S$$

where* K*_*p*_ is the proportional gain; *K*_*i*_ is the integral gain; *K*_*d*_ is the differential gain. *e(h)* is the control error; *T*_*i*_ is integral time constant; *T*_*d*_ is differential time constant.

*e(h)* is as follows:

42 $$e(h) = r(h) - y(h)$$

Expression for the discretization of the derivative and integral terms in Eq. (40) is given by:

43 $$u(m) = K_{p} e(m) + K_{i} \sum\limits_{j = 0}^{m} e (j)TuK_{d} \frac{e(m) - e(m - 1)}{{Tu}}$$

where* T*_*u*_ is time.

The integral absolute value of error (IAE) is used to calculate the error:

44 $$J_{1} = {\text{IAE}} = \int_{0}^{\infty } {|e} (h)|{\text{d}}h$$

### Standard particle swarm algorithm

To optimize the performance of the entire control system and achieve the ideal value for the controller's objectives, coordinate the parameters accordingly. Apply a particle swarm algorithm (PSO) to optimize these three parameters, seeking the most appropriate values within the specified range^37^.

Standard particle swarm algorithm is a mature swarm intelligence algorithm inspired by foraging scenarios observed in bird flocks and bee colonies^38^. It treats individual organisms as particles, searching in a D-dimensional space, iteratively seeking the optimal solution^39^. The algorithm evaluates results based on fitness and ultimately searches for the global optimum based on the best solution found during the current search^40^. Throughout the iteration process, a particle's motion consists of velocity and position, where the particle's velocity is determined by inertia, self-cognition, and social experience^41^. The optimization equations for the particle swarm are expressed in Eqs. (45) and (46).

45 $$v_{ij} (t + 1) = wv_{ij} (t) + c_{3} r_{1} \left[ {\left( {p_{ij} } \right)_{j} (t) - x_{ij} (t)} \right] + c_{4} r_{2} \left[ {\left( {p_{gj} } \right)_{j} (t) - x_{ij} (t)} \right]$$

46 $$x_{ij} (t + 1) = x_{ij} (t) + v_{ij} (t + 1)$$

where *j* indicates the particle's *jth* dimension;* i* stands for particle* i*; *t* indicates the number of iterations; *c*_*3*_, *c*_*4*_ is the acceleration factor; *r*_*1*_ and* r*_*2*_ are mutually independent stochastic functions (with values between 0 and 1); *w* is the inertia weight parameter (with values between 0 and 2). Among them, the initial velocity and position of particles are randomly generated, and then iterated continuously according to the formula.

The velocity update of a particle consists of three parts in above equation: the first part, known as the inertia factor, reflects the influence of the particle's current velocity on its direction and speed, playing a role in both local search and balancing global exploration capability; the second part represents the particle's self-awareness, allowing it to explore various points within the search space based on its own memory; and the third part involves interactions among particles, thereby enables the PSO population to collaboratively search for the optimal solution. This highlights the particle swarm optimization algorithm's strong global search capabilities.

### Improved particle swarm algorithm

When standard particle swarm algorithm operates for a certain period, particles lose diversity among themselves and tend to hover around local optima. This susceptibility to converging to local optima can lead to stagnation in the overall iteration of the population. The particles cease exploring other regions, ultimately hindering the discovery of the global optimum^42^.

To tackle the inherent characteristics of PSO, two primary directions for algorithm improvement are identified. One approach involves enhancing algorithmic parameters, with a particular focus on the inertia weight parameter (*w*). The value of *w* plays a crucial role in balancing global and local searches. Some researchers have suggested modifications to the inertia weight parameter^43^. In this context, particles engage in global exploration during the initial stages and transition to local refinement post-convergence to attain precise values. The second direction revolves around augmenting the diversity of the population. By integrating genetic algorithms and introducing velocity mutation, position mutation, and other techniques, the diversity of particles can be increased, thereby enhancing global search capability^44^. These proposed solutions effectively address the challenges associated with global search difficulty. However, it remains crucial to strike a balance between global exploration and convergence speed for optimal algorithmic performance.

Moreover, Research in particle swarm optimization suggests that the optimal particle (*p*_*g*_), which is identified based on the performance of the particle swarm in the search space, is typically located near the global optimum. This optimal particle may vary during each iteration of the algorithm. Equation (45) reveals that the velocity update formula simplifies to only the first term. When the inertia weight is excessively large, the current *p*_*g*_ may overshoot its neighborhood, potentially missing the true global optimum. Therefore, an effective search strategy involves particles closer to *p*_*g*_ having smaller velocities, facilitating a fine-grained search within the neighborhood of the optimal particle.

This study introduces a similarity-based approach to dynamically adjust the inertia weight during iterations, relying on the similarity between each particle and the optimal particle. This ensures that each particle possesses a different *w* value at each iteration. As PSO iterations progress, particles gradually converge toward the optimal point, resulting in increased similarity among particles and a higher likelihood of falling into local optima. The similarity between two particles *i* and *j* is defined as follows:

*s (i, j)* = 0 when *d (i, j)* → ∞; 0 ≤ *s (i, j)* ≤ 1 for any particle.

The following equation is defined to calculate the similarity of *i* and *j*:

47 $$s(i,j) = \left\{ {\begin{array}{*{20}c} {1,} & {d(i,j) < d_{\min } } \\ {1 - \left[ {\frac{d(i,j)}{{d_{\max } }}} \right]} & {d_{\min } \le d(i,j) < d_{\max } } \\ {0,} & {d(i,j) \ge d_{\max } } \\ \end{array} } \right.$$

where *d (i, j)* is expressed in terms of their Euclidean distance, and *d*_*max*_ and *d*_*min*_ are determined based on the search region of the particle.

From the iterative equation, it's evident that as the optimization process nears the optimal point, the impact of *w* on the flight velocity becomes more pronounced. To enable a fine-grained search within the vicinity of the optimal solution, particles near the optimum should be assigned smaller *w* values. This implies that particles bearing higher similarity to the current optimal particle should be assigned smaller *w* values.

The formula for calculating of *w* is as follows:

48 $$w_{i} = w_{\max } - s(i,j)\left( {w_{\max } - w_{\min } } \right)$$

49 $$w_{i} = w_{\min } + \left( {w_{i} - w_{\min } } \right) \times \frac{{t_{\max } - t}}{{t_{\max } }}$$

The particle's *w* decreases both linearly with the number of iterations and as the particle's resemblance to the ideal particle rises, as indicated by Eqs. (48) and (49). This algorithm is beneficial for global search because it initiates the iteration with a large weight value that progressively decreases toward the end, demonstrating good convergence ability. Moreover, the inertia weights of different particles vary; those close to the optimal particle (*p*_*g*_) have reduced inertia weights. This implies that particles near *p*_*g*_ focus more on fine search than global search, increasing the probability of discovering the true global optimal solution.

**Figure 2:**
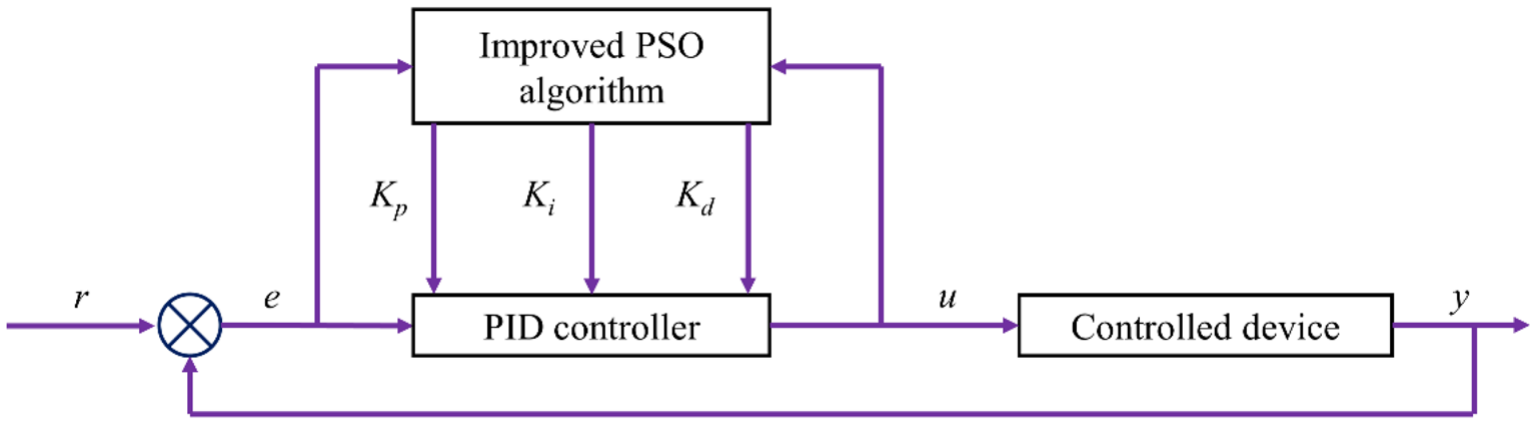
Structure of the improved PSO-PID control.

**Figure 3:**
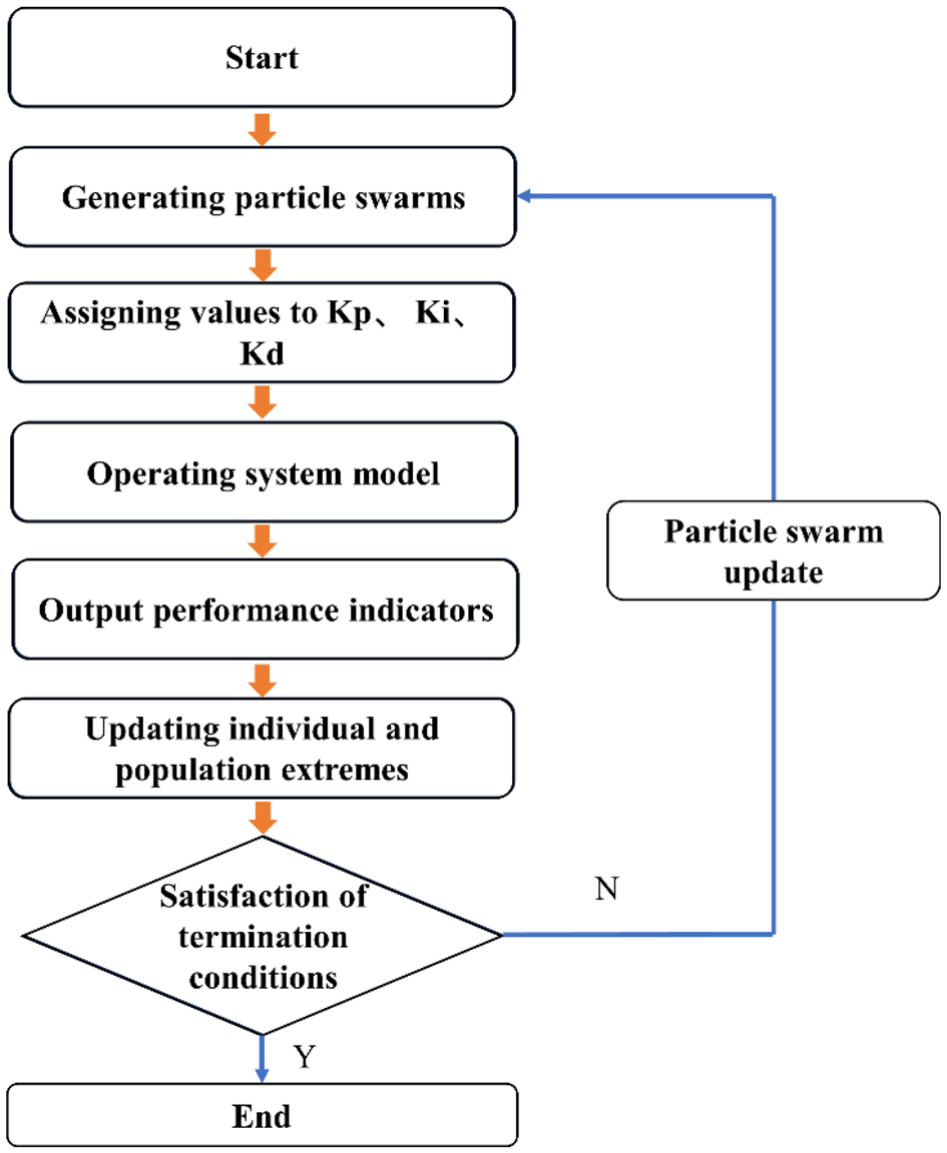
Flowchart of the improved PSO-PID control.

Figure 2 illustrates the structure of the improved PSO-PID control system. The fundamental concept behind PSO-PID parameter optimization involves creating a model for optimizing PID controller parameters using an improved particle swarm algorithm. In this model, the three parameters of PID (proportional, integral, and differential) are amalgamated as particles within the particle swarm. During the optimization process, each particle iteratively adjusts its speed and position based on its individual experiences and collective group experiences, gradually converging towards the optimal objective and ultimately achieving the desired outcome. The flowchart of the improved PSO-PID control system is presented in Fig. 3.

## Results and discussion

### Dual-fuel engine model validation

**Figure 4:**
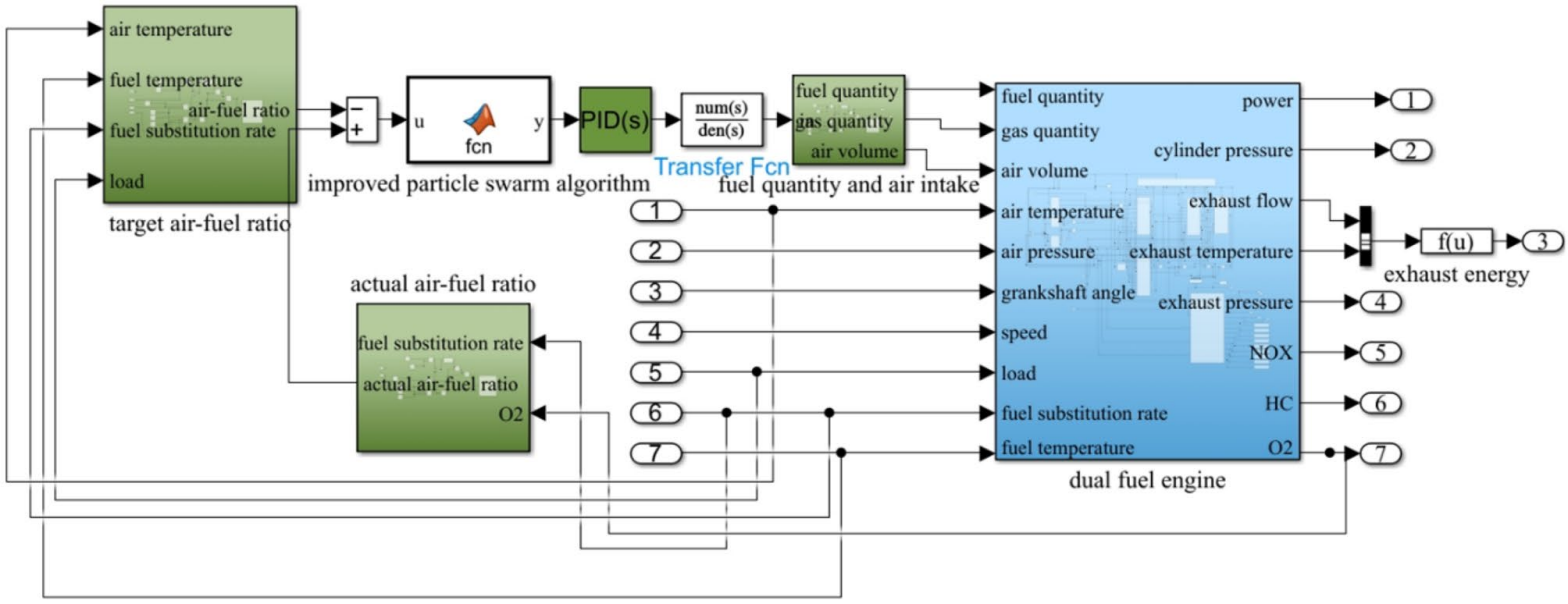
Dual-fuel engine simulation model.

The simulation model of the dual-fuel engine is constructed using Matlab/Simulink, incorporating the mathematical relationships described above as shown in Fig. 4.

**Figure 5:**
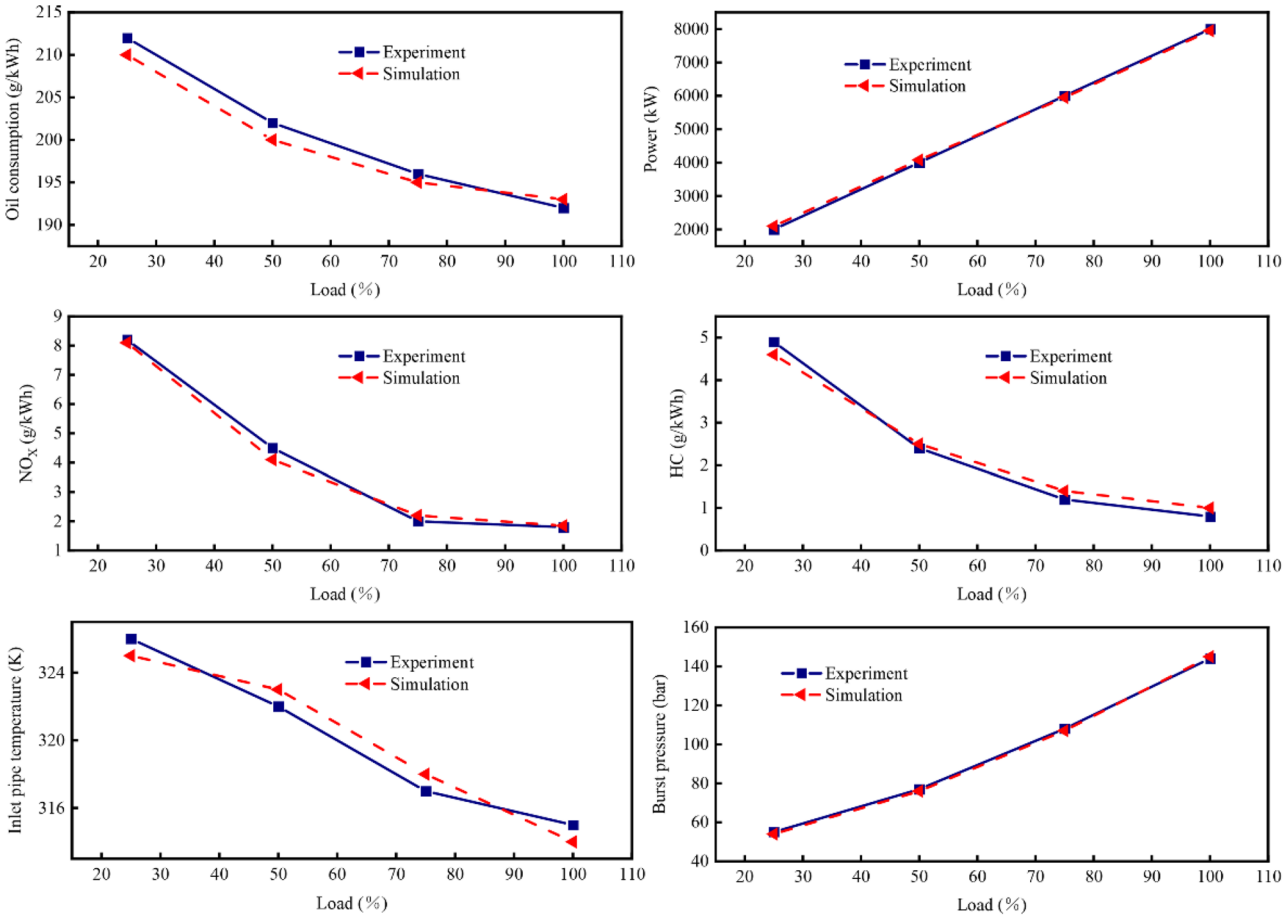
Model validation in diesel mode.

**Figure 6:**
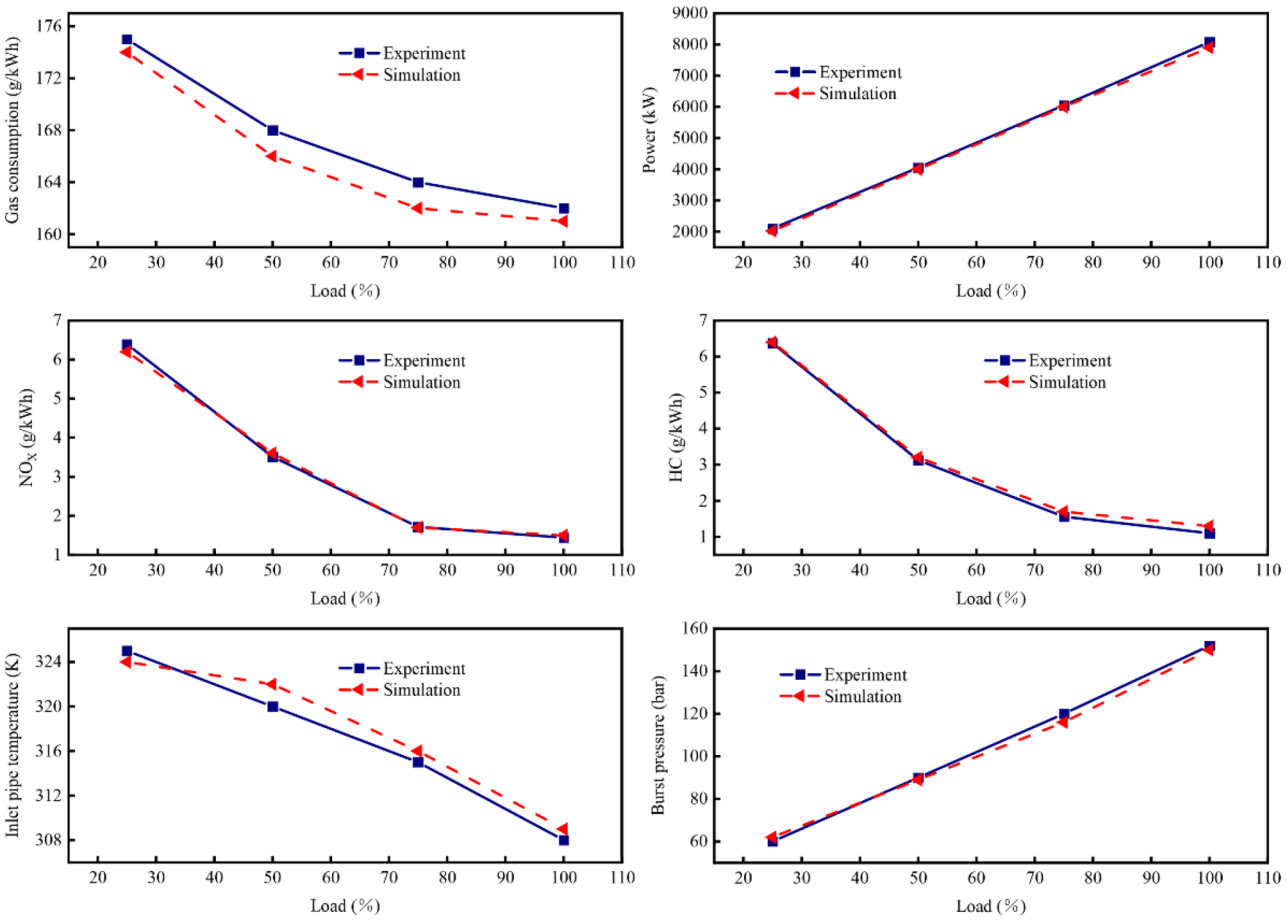
Model validation in gas mode.

Figures 5 and 6 present a comparison of the main parameters in the diesel mode and gas mode of the dual-fuel engine between simulation and experiment. Notably, key performance indicators reflecting the engine's performance exhibit a close fit between simulation and experiment, the maximum error was 2.51% for the diesel mode and 2.83% for the gas mode, with an error of less than 3%. This suggests that the established dual-fuel engine model is precisely calibrated.

### Steady-state and dynamic characteristics of dual-fuel engine

**Figure 7:**
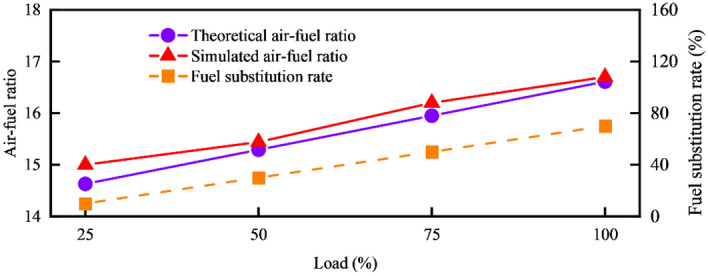
Comparison of simulated and theoretical air–fuel ratios.

The air–fuel ratio in a dual-fuel engine significantly impacts its overall performance. Based on the theoretical calculations discussed earlier, the target air–fuel ratio for the liquefied natural gas/diesel dual-fuel engine studied in this paper needs to be configured according to different operating conditions. Under low-load conditions, where the compressor intake is limited, and combustion is insufficient, the air–fuel ratio is kept small but not less than 15. This helps reduce the engine's equivalent fuel consumption rate and emissions of pollutants such as NO_X_ and HC. In the middle load region, the optimal air–fuel ratio falls between 15 and 16, contributing to better overall engine performance, in the high-load condition, the air–fuel ratio should not be a larger value, but it should not be less than 16. Figure 7 illustrates the comparison between the target air–fuel ratio and the theoretical air–fuel ratio for the four operating conditions.

**Figure 8:**
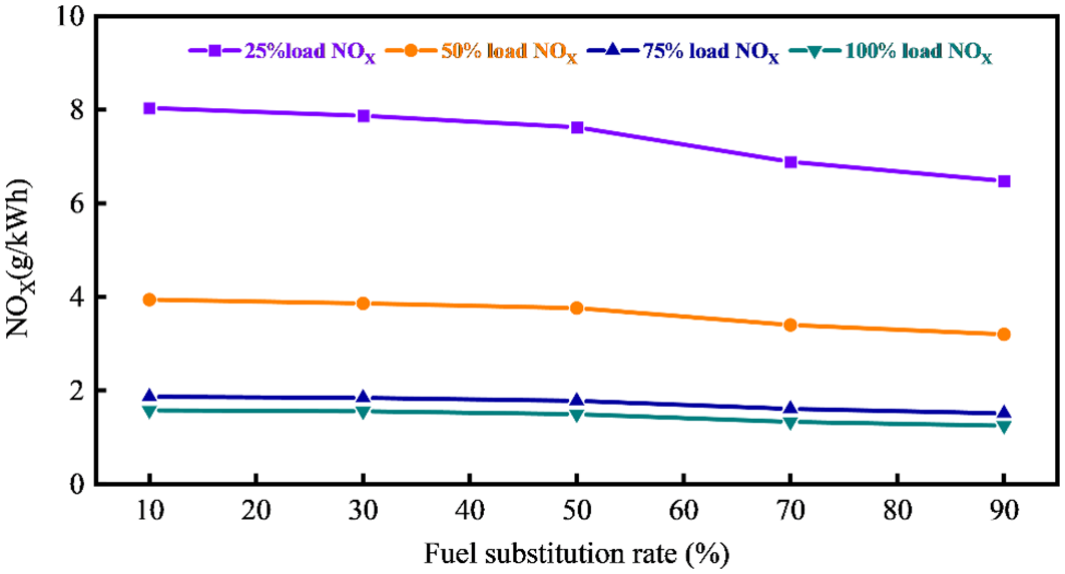
Variation of NO_X_ at different loads and fuel substitution rates.

**Figure 9:**
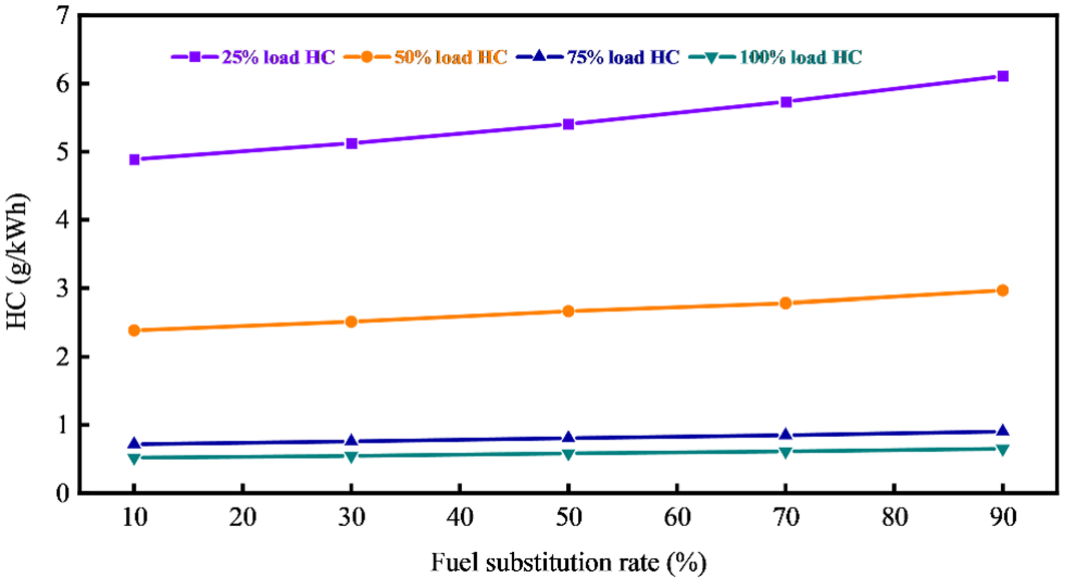
Variation of HC under different loads and fuel substitution rates.

Figures 8 and 9 illustrate the variations in NO_X_ and HC under different loads and fuel substitution rates. Notably, a decrease in the fuel substitution rate correlates with a continuous rise in the specific emissions of NO_X_. This trend is primarily attributed to the reduced natural gas injection quantity, causing an overall decrease in the specific heat capacity of the cylinder due to the larger specific heat capacity of natural gas. Additionally, a higher proportion of diesel in the mixture with natural gas/air expands the ignition area of diesel, promoting complete combustion and elevating the combustion temperature within the cylinder, thus facilitating NO_X_ formation. Furthermore, the reduction in natural gas injection increases the fresh air volume entering the cylinder, expanding the oxygen-rich area and favoring NO_X_ synthesis.

Conversely, at higher fuel substitution rates, where diesel/natural gas/air premixed combustion occurs within the cylinder, the high equivalence ratio of natural gas and a smaller diesel injection mass result in a lower release of heat during the diesel diffusion combustion stage. Consequently, the cylinder temperature does not rise rapidly, leading to a reduction in nitrogen oxide emissions.

However, reducing the fuel substitution rate significantly decreases HC emissions. This is because a larger quantity of natural gas enters the cylinder, increasing the concentration area of gases within the cylinder, worsening the combustion conditions and causing incomplete combustion. Simultaneously, the low temperature and pressure within the cylinder result in poor diesel atomization, uneven mixing of diesel/natural gas/air, and suppression of complete combustion of natural gas. When the injection volume of natural gas decreases, the equivalence ratio of natural gas decreases. The temperature and pressure within the cylinder continually rise, and the increased diesel injection mass raises the heat release, promoting the complete combustion of natural gas and leading to a decrease in HC emissions.

**Figure 10:**
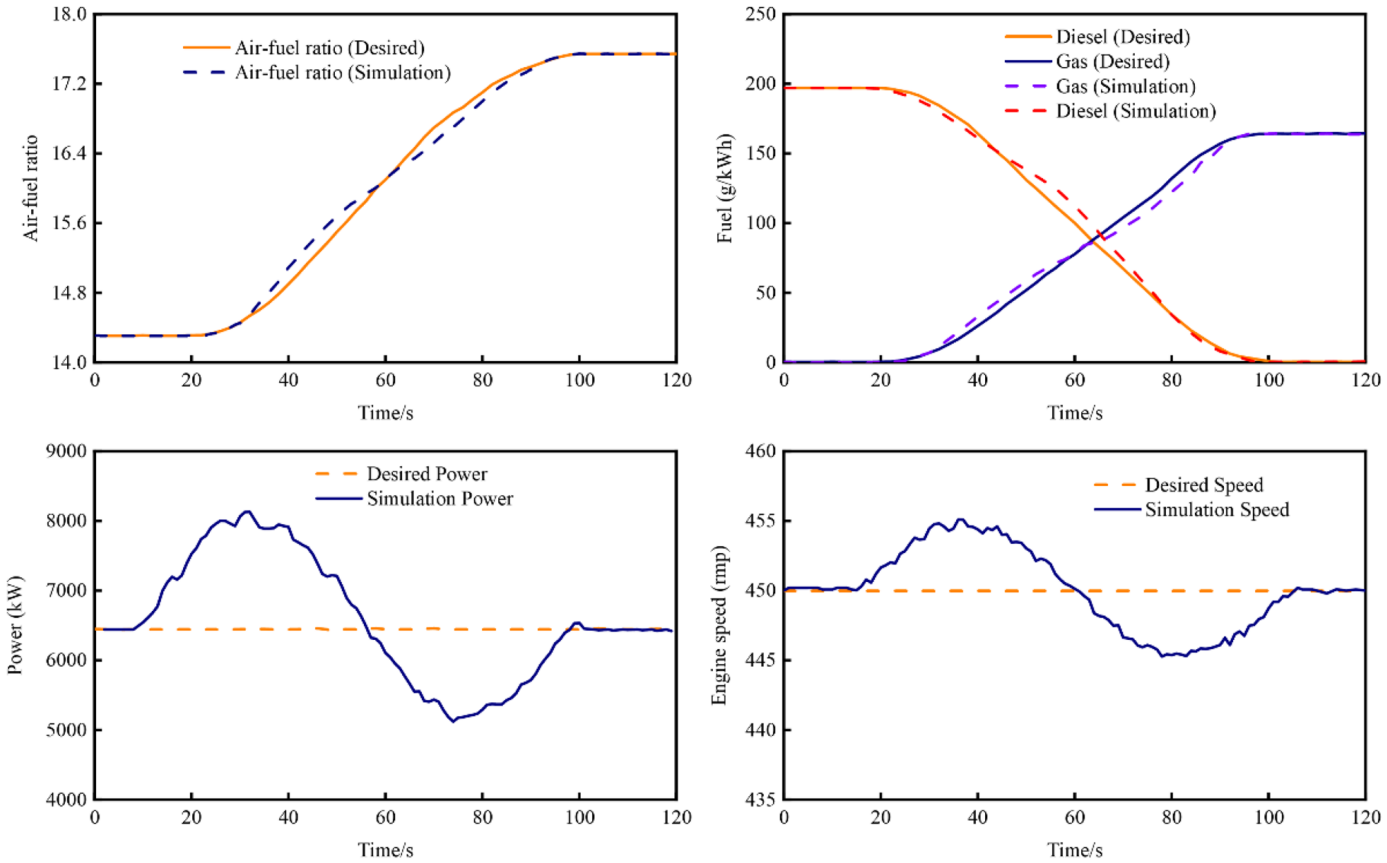
The response curve of engine performance parameters.

According to the engine operation manual, dual-fuel engines possess the capability to transiently switch from diesel mode to gas mode when the engine load is below 80%, ensuring the safe operation of the engine. Moreover, they can seamlessly switch from gas mode to diesel mode under any load condition. This study specifically focuses on the transition from diesel mode to gas mode at an 80% load. Figure 10 illustrates the transient process of the engine performance parameters' response curve. As can be seen, in the transition from diesel mode to gas mode, the entire fuel conversion process is completed within 2 min. Throughout this transition, the air–fuel ratio and fuel consumption exhibit minimal deviation from the desired values. However, the engine's power and speed display more noticeable fluctuations. The power fluctuates within the range of 5000–8000 kW, while the speed fluctuates in the range of 445–455 rpm. Despite these fluctuations, the developed control system described in this paper proves effective in realizing the control of engine operating conditions.

### Comparison of control performance

#### Control performance under different response patterns

**Figure 11:**
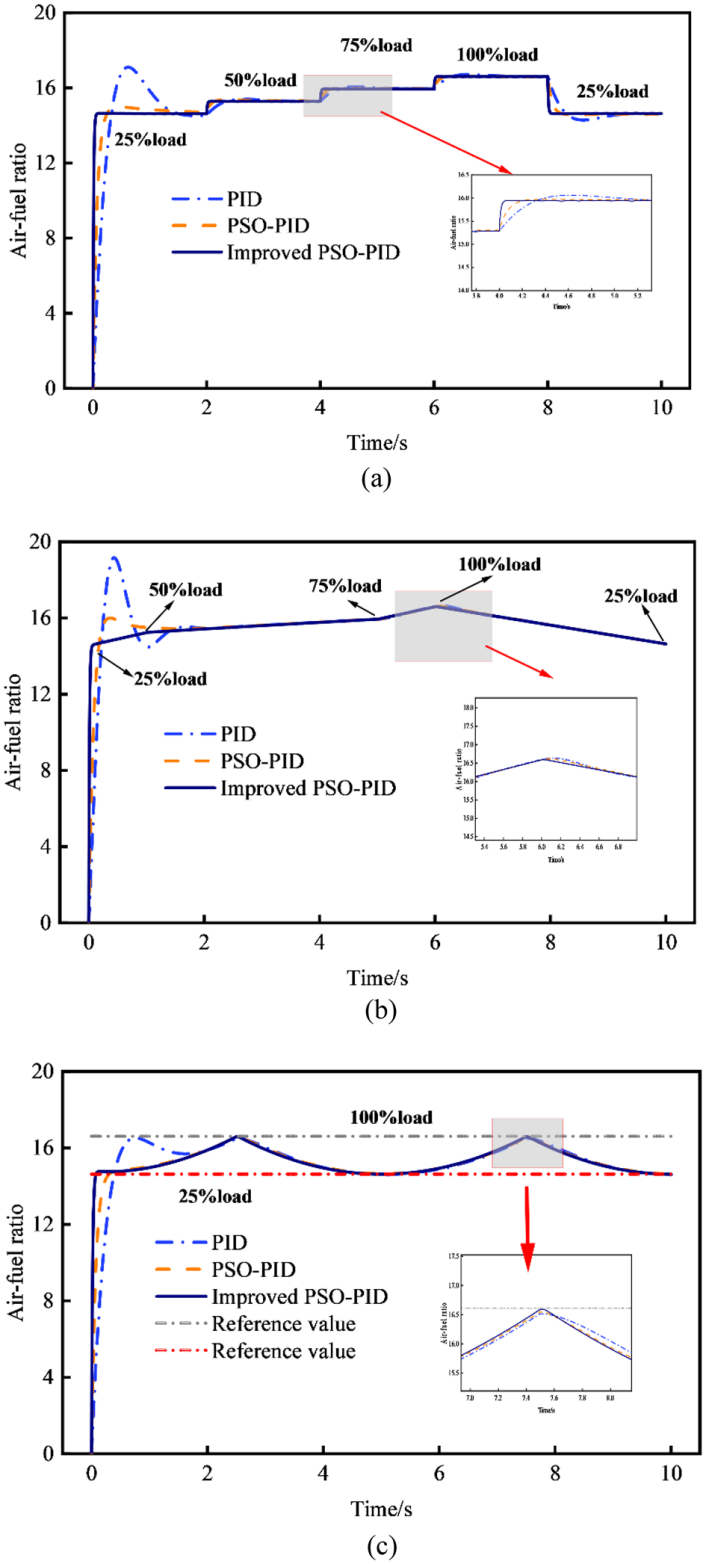
Comparison of different controls in response modes with different air–fuel ratios. (**a**) Comparison of step change response. (**b**) Comparison of continuous change to node change response. (**c**) Comparison of periodic change response.

The control performance of air–fuel ratios under various response patterns is depicted in Fig. 11 for traditional PID control, PSO-PID control, and improved PSO-PID control. These patterns include step response, which simulates a sudden change in load; continuous change to node change response, which simulates a gradual and continuous increase or decrease in engine load; and periodic change response, which mimics the periodic load changes experienced by a marine dual-fuel engine on a fixed route. In the improved PSO algorithm, parameters *c*_*3*_ and *c*_*4*_ are set to 1.495. The maximum inertia weight is 1.5, and the minimum inertia weight is 0.4. The number of iteration steps is 60.

The response control simulation is conducted under the scenarios include 25% load with 10% fuel substitution, 50% load with 30% fuel substitution, 75% load with 50% fuel substitution, and 100% load with 70% fuel substitution. As can be seen, the improved PSO-PID control exhibits superior performance in terms of convergence speed and stability compared to traditional PID and PSO-PID across various response modes, ensuring the system's responsiveness. Notably, it excels in controlling step and nodal variations in the air–fuel ratio, displaying greater accuracy and stability in smooth cyclic variations compared to traditional PID and PSO-PID controls. Using the step response of air–fuel ratio as an illustration, the improved PSO-PID controller exhibits notable improvements over both the traditional PID and PSO-PID controllers. Specifically, compared to the traditional PID controller, the improved PSO-PID controller reduces the response time by 0.47 s and the maximum overshoot by 98.43%, while decreasing the absolute errors by 87.42%. Similarly, compared to the PSO-PID controller, the improved PSO-PID controller reduces the response time by 0.21 s, the maximum overshoot by 96.05%, and the absolute errors by 90.55%. Table 7 shows the comparison of PID setting parameters and performance indicators under different control methods and response modes.

**Table 7:** Comparison of setting parameters and performance indicators.

Response mode	Parameters	PID	PSO-PID	Improvd PSO-PID
Step change response	*K*_*p*_	1.732	1.401	1.214
	*K*_*i*_	0.378	0.592	0.914
	*K*_*d*_	8.125	12.013	13.765
	Time response (s)	0.65	0.39	0.18
	Overshoot (%)	17.45	2.82	0.61
	Iterations	–	80	55
	IAE	9.51	4.27	1.36
Continuous change to node change response	*K*_*p*_	1.854	1.576	1.439
	*K*_*i*_	0.412	0.633	0.927
	*K*_*d*_	8.534	12.815	13.926
	Time response (s)	0.51	0.45	0.15
	Overshoot (%)	29.53	6.97	0.47
	Iterations	–	88	59
	IAE	8.21	4.31	1.29
Periodic change response	*K*_*p*_	1.791	1.423	1.294
	*K*_*i*_	0.427	0.645	0.919
	*K*_*d*_	8.312	12.225	13.512
	Time response (s)	0.70	0.44	0.16
	Overshoot (%)	10.07	1.11	0.25
	Iterations	–	95	57
	IAE	6.84	1.92	1.15

**Figure 12:**
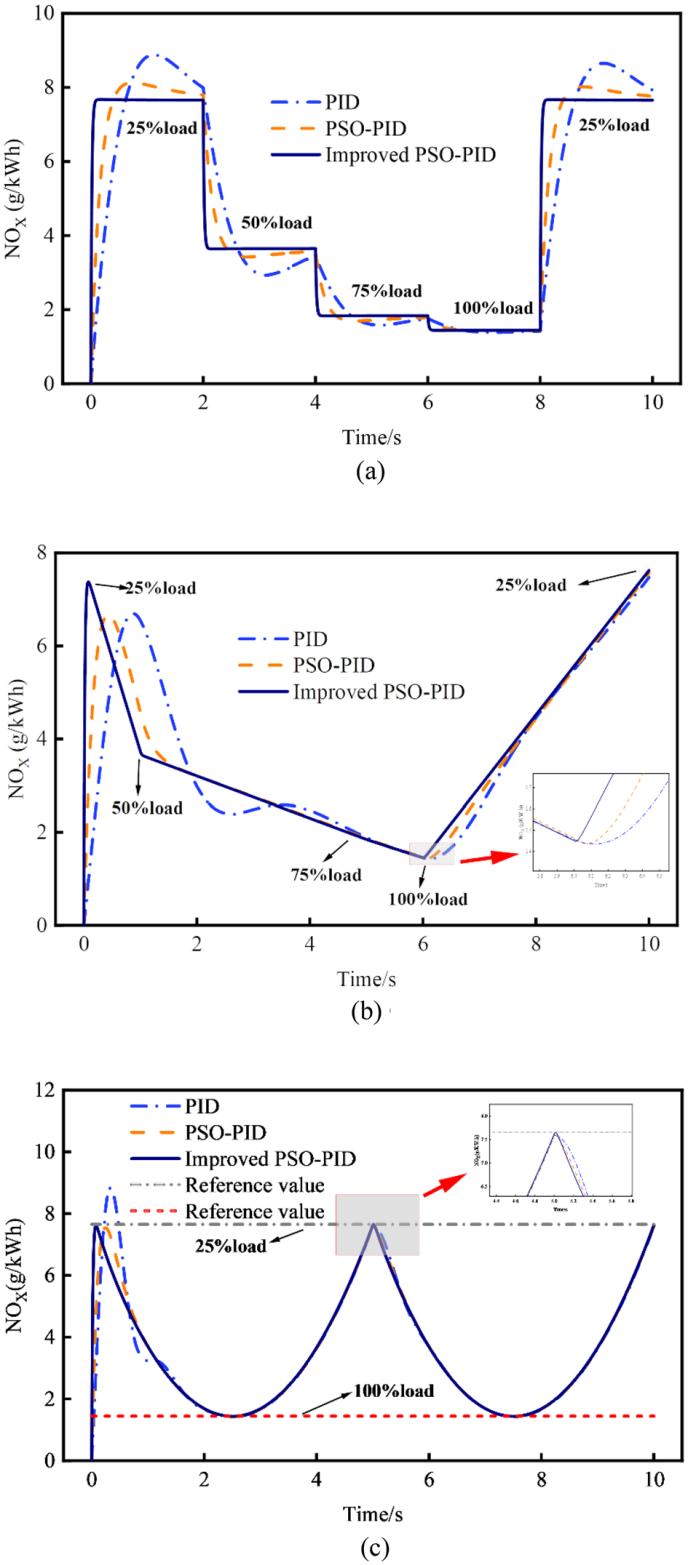
Comparison of NO_X_ emissions control performance in various response modes. (**a**) Comparison of step change response. (**b**) Comparison of continuous change to node change response. (**c**) Comparison of periodic change response.

**Figure 13:**
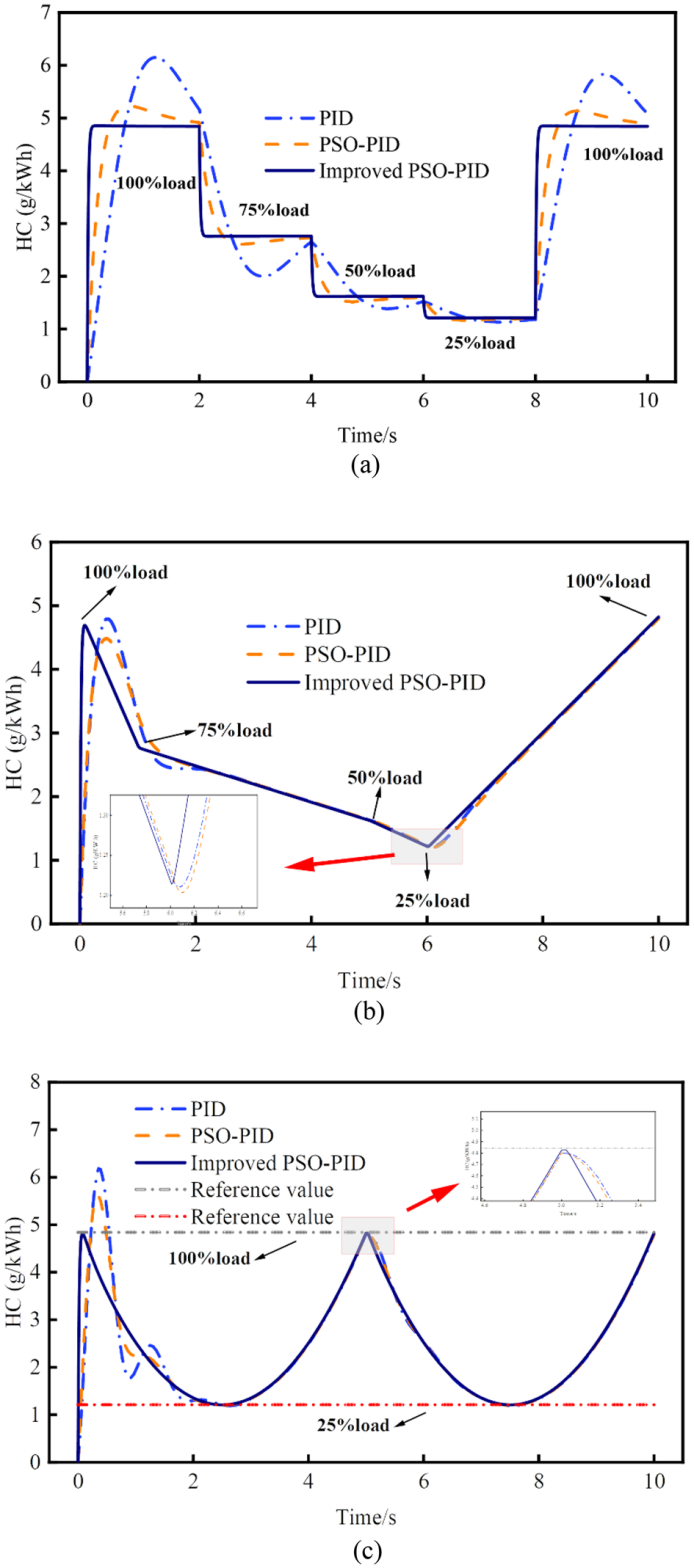
Comparison of HC emissions control performance in various response modes. (**a**) Comparison of step change response. (**b**) Comparison of continuous change to node change response. (**c**) Comparison of periodic change response.

Figures 12 and 13 provide a comprehensive view of the impact of traditional PID control, PSO-PID control, and improved PSO-PID control on NO_X_ and HC emissions across various operational conditions and response modes. Notably, traditional PID control exhibits a larger overshoot and requires a longer time to stabilize, as seen in both NO_X_ and HC emissions. Conversely, the improved PSO-PID control demonstrates superior tracking performance, showcasing its effectiveness in promptly and accurately responding to dynamic changes in operating conditions. For example, considering the step response of NO_X_ emissions, compared to the traditional PID and PSO-PID controllers, the improved PSO-PID reduces the response time by 1.15 s and 0.83 s, respectively. Moreover, the maximum overshoot decreases by 98.91% and 97.68%, while the absolute errors reduce by 89.31% and 91.75%, respectively.

**Figure 14:**
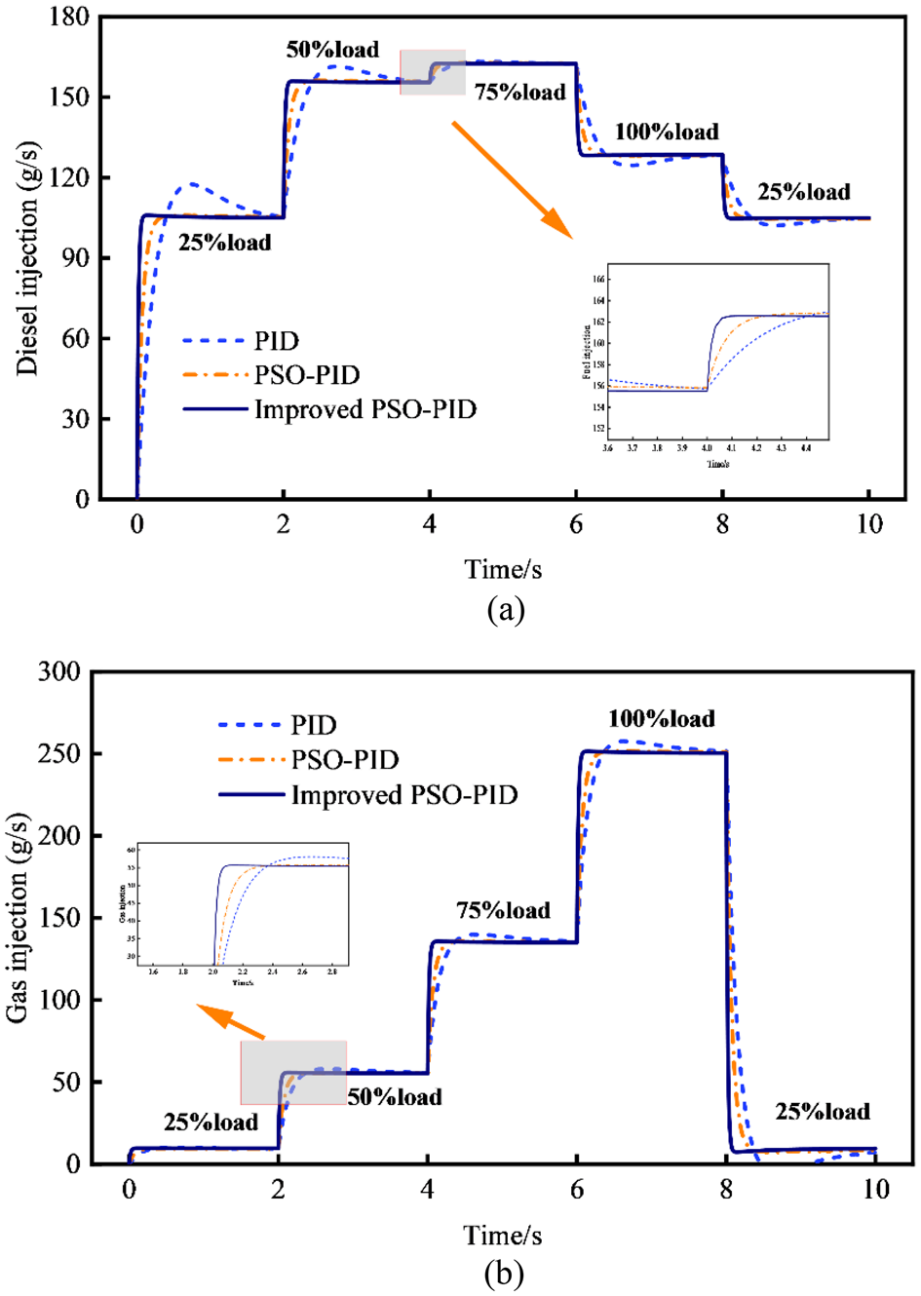
Step-by-step reaction of natural gas injection volume and diesel injection volume. (**a**) Volume of diesel injection. (**b**) Volume of natural gas injection.

Figure 14 presents a comparison of the effects of natural gas and diesel injection volumes under various operating situations. Notably, improved PSO-PID control demonstrates significant enhancements in reducing overshoot and improving stability compared to traditional PID control and PSO-PID control. Particularly depicted in Fig. 14a, compared to the traditional PID and PSO-PID methods, the improved PSO-PID control demonstrates a significant reduction in maximum overshoot by 97.21% and 95.79%, respectively. Additionally, it achieves a shortened response time of 0.68 s and 0.22 s, respectively. Furthermore, the absolute errors were reduced by 90.02% and 89.39%, respectively.

#### Control performance under mode switching

**Figure 15:**
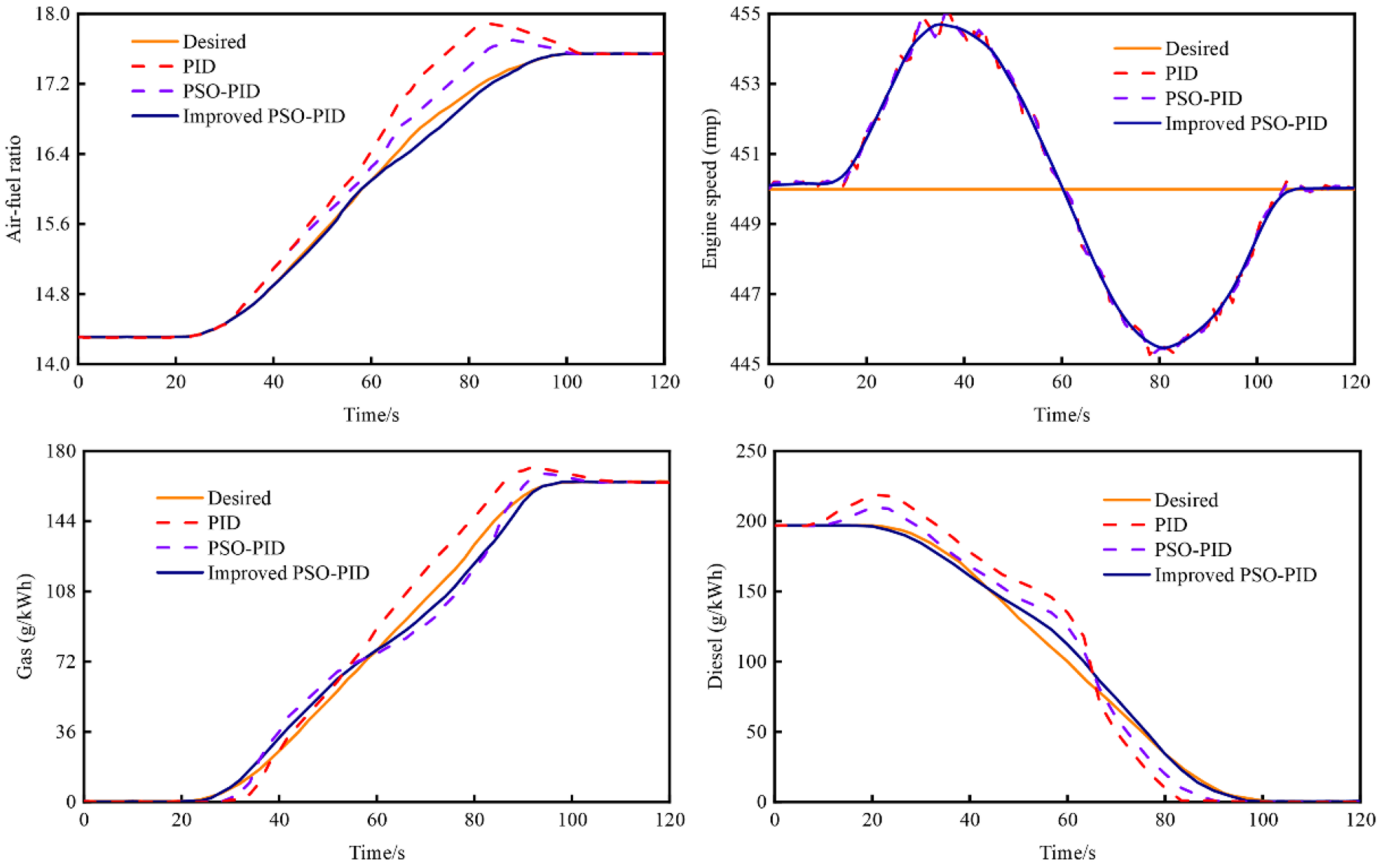
The comparison of control effects during mode transitions.

In addition to load changes, mode switching is also a common operation for dual-fuel engines, which can achieve different emissions, different power requirements. Table 8 shows the comparison of set parameters and performance indexes of the air–fuel ratio under different controls during the mode switching of the dual fuel engine. The stability and response time of the mode switching have an important effect on the operation of the system. Figure 15 provides a comparative analysis of control effects during the engine's transition from diesel mode to gas mode at an 80% load. The illustration clearly indicates that, while the three control methods exhibit similar effects on engine speed control, the improved PSO-PID control method surpasses both PID and PSO-PID controls in terms of air–fuel ratio and specific fuel consumption control, showcasing a superior control effect. Taking air–fuel ratio control as an example, the improved PSO-PID control method demonstrates superior performance compared to traditional PID and PSO-PID methods. The response time is reduced by 5.95 s and 5.17 s, respectively; the maximum overshoot is decreased by 96.36% and 95.29%, respectively; and the absolute errors are reduced by 88.22% and 90.09%, respectively.

**Table 8:** Comparison of setting parameters and performance indexes under air fuel ratio control.

Parameters	PID	PSO-PID	Improvd PSO-PID
*K*_*p*_	2.267	2.107	1.975
*K*_*i*_	0.519	0.682	0.810
*K*_*d*_	7.923	8.534	11.226
Time response (s)	33.51	32.78	27.56
Overshoot (%)	5.23	3.07	1.25
Iterations	–	100	58
IAE	34.33	20.02	4.85

## Conclusion

A comprehensive thermodynamic model and functional model of a large marine dual-fuel engine were developed in the Matlab/Simulink environment. An improved particle swarm optimization (PSO) algorithm was applied to this simulation model to evaluate its control effectiveness and compared with traditional PID and PSO-PID control methods. Through the analysis of the results, the following conclusions are drawn:

1. The developed model was validated against experimental data, and the results showed good agreement, with an average deviation of less than 3%. The simulations demonstrated that the model can accurately predict fuel consumption, power output, and pollutant emissions.
2. Dual-fuel engines possess the capability to transiently switch from diesel mode to gas mode, completing the entire fuel conversion process within 2 min when the engine load is below 80%. They can also seamlessly switch from gas mode to diesel mode under any load condition.
3. Three different response modes are used to simplify the specific working conditions of dual fuel engine. The improved PSO-PID controller reduces the response time by 0.47 s and 0.21 s, the maximum overshoot by 98.43% and 96.05%, and decreases the absolute errors by 87.42% and 90.55%, respectively, in air–fuel ratio control using the step response method compared with traditional PID and PSO-PID controllers.
4. In the context of mode switching, the improved PSO-PID control demonstrates significantly enhanced performance compared to traditional PID and PSO-PID control methods. Specifically, the response time in air–fuel ratio control is reduced by 5.95 s and 5.17 s, respectively. Additionally, the maximum overshoot is decreased by 96.36% and 95.29%, respectively, and the absolute errors are reduced by 88.22% and 90.09%, respectively.

## Data Availability

The authors would like to confirm that all data generated or analysed during this study are included in this published article.
